# σ‐Resonance Stabilization of Aminomethylene by *N*‐Halogenation

**DOI:** 10.1002/jcc.70360

**Published:** 2026-03-29

**Authors:** Serhii Medvedko, Virinder Bhagat, J. Philipp Wagner

**Affiliations:** ^1^ Institut für Organische und Analytische Chemie, Universität Bremen Bremen Germany; ^2^ Institut für Orgainische Chemie, Eberhard Karls Universität Tübingen Tübingen Germany

**Keywords:** *N*‐heterocyclic carbenes, reactive intermediates, resonance stabilization

## Abstract

Amino substitution effectively stabilizes carbenes via π‐resonance, enabling their isolation as *N*‐heterocyclic carbenes (NHCs). Our group has recently demonstrated that *N*‐halogenation of pyridinylidenes (Hammick intermediates) provides an additional stabilization mode through a rarely utilized σ‐resonance. However, this σ‐resonance interaction might not yet have reached its full potential due to the constriction of the carbene motif within a cyclic structure. Thus, we have studied the impact of *N*‐halogenation (X = Br, I) on the structure, energetics, and spectroscopic properties of the parent open‐chain aminocarbene, aminomethylene, utilizing fully unconstrained CCSD(T) and NEVPT2 geometry optimizations together with a def2‐TZVPP basis set. We find that the halogenated carbenes prefer a *Z* configuration of the C–N bond, which is impossible in a cyclic arrangement. Halogenated aminomethylenes are kinetically protected against unimolecular rearrangement by barriers exceeding 35 kcal mol^−1^. Short C–N bond lengths (1.23 Å) and wide carbene angles (~125°) indicate cooperative π‐ and σ‐resonance stabilization, resulting in carbene stabilization energies (CSEs) increased by ~12–13 kcal mol^−1^ relative to the parent aminomethylene. The electronic structure is characterized by the σ* orbital of the N–X bond as the lowest unoccupied molecular orbital.

## Graphical abstract

Utilizing CCSD(T) and NEVPT2 optimizations, we demonstrate that the simplest aminocarbene, aminomethylene, gets further stabilized upon halogen substitution of its nitrogen atom. The so achieved σ‐resonance stabilization, evident in a shortening of the C–N bond length and widening of the carbene angle, results in an increased carbene stabilization energy relative to parent aminomethylene.

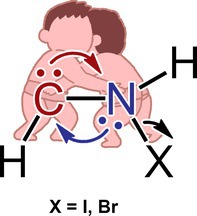

## 1 Introduction

The isolation of stable carbenes has been a longstanding goal in chemistry, dating back at least to 1835, when Dumas attempted to generate methylene (CH_2_) by dehydration of methanol [1]. Much later, Wanzlick recognized that carbenes benefit from diamino substitution and postulated the existence of a carbene dimerization equilibrium [2], a notion that later proved to be system‐dependent [3, 4, 5] and was refuted by Lemal for the carbene originally investigated [6]. A decisive breakthrough occurred in 1991 with Arduengo's isolation of the first stable, crystalline *N*‐heterocyclic carbene (NHC) [7]. In these systems, the electronegative nitrogen atoms stabilize the carbene center by lowering the energy of the lone pair, while π‐donation from the nitrogen lone pairs raises the energy of the vacant p orbital at the carbene carbon atom. This results in a substantial thermodynamic stabilization, enabling the isolation of singlet ground‐state carbenes [8, 9]. Today, both monoamino (CAACs) [10, 11] and diamino carbenes are widely employed and play central roles in catalysis as well as in transition metal and main group chemistry [8, 9, 12, 13].

**FIGURE 1:**
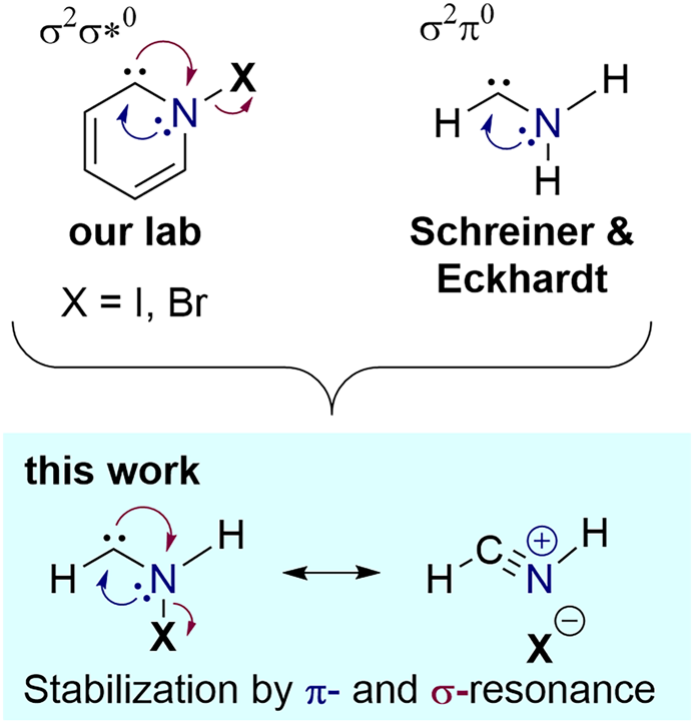
*N*‐Halogenated pyridinylidenes are stabilized by π‐ and σ‐resonance interaction. Introduction of *N*‐halogenation into the open‐chain structure of aminomethylene possibly further enhances this effect.

In a serendipitous discovery, our lab recently identified an entirely new class of carbenes during studies on the photolysis of 2‐halopyridines in cryogenic noble gas matrices. The thus generated *N*‐iodo [14] and *N*‐bromo pyridinylidenes [15] (Figure 1) are additionally stabilized by σ‐resonance [16, 17] arising from the interaction of the carbene lone pair with the antibonding σ*_N–X_ orbital, which are coplanar. If the N–X bond is sufficiently weak (X = I, Br, Cl), this interaction becomes so significant that the geometric and electronic structure of the carbene changes. While the highest occupied molecular orbital (HOMO) remains the σ‐type lone pair at the carbene center, the lowest unoccupied molecular orbital (LUMO) is no longer π‐type but instead corresponds to the σ*_N–X_, classifying these species as σ^2^σ*^0^ carbenes (as opposed to the σ^2^π^0^ configuration) [14, 15, 18]. The resonance gives rise to characteristic spectral features of these carbenes, including strongly blue‐shifted C–N stretching vibrations in the IR spectrum above 1650 cm^−1^ and a strong UV/vis absorption band around 400 nm, corresponding to the σ–σ* transition [14]. Furthermore, the preferred reaction path with molecular hydrogen shifts from an out‐of‐plane geminal di‐hydrogenation reaction [19, 20] to a sideways addition, producing pyridinium halide [14, 18].

Despite the successful stabilization of pyridinylidenes by σ‐resonance, which is otherwise only possible when utilizing excessive steric protection [21, 22], we reasoned that this stabilization mode has not yet reached its full potential due to the geometric constraints imposed by the cyclic framework. Hence, we set out to study an open‐chain amino‐substituted carbene [23, 24] that does not suffer from this limitation. In this context, it is particularly notable that Schreiner and Eckhardt recently isolated the parent aminocarbene, aminomethylene, in an inert gas matrix by pyrolysis of cyclopropylamine, further confirming that even a single amino group provides significant stabilization through π‐resonance (Figure 1) [25]. We, therefore, sought to study the impact of *N*‐halogenation (X = I, Br) on the structural, energetic, and spectroscopic properties of this compound.

## 2 Results and Discussion

We have optimized *N*‐iodo‐ (**1**) and *N*‐bromoaminomethylene (**2**) as well as their relevant isomers and the interconnecting transition states at the CCSD(T)/def2‐TZVPP level of theory (Figures S1–S26), subsequently refining the electronic energy by extrapolation to the complete basis set (CBS) limit. The resulting potential energy surfaces are depicted in Figure 2. As expected for aminocarbenes, **1** and **2** possess singlet ground states with their triplet excited states being 17.9 and 23.8 kcal mol^−1^ higher in energy, respectively. While the halogen atoms exhibit significant binding in the singlet state, the triplet structures acquire characteristics of a radical pair with spin densities on the carbon and halogen atoms near unity. This is in agreement with the computed N–X bond dissociation energies (BDEs; cf. Figure 2), which are somewhat higher than the singlet–triplet gaps.

**FIGURE 2:**
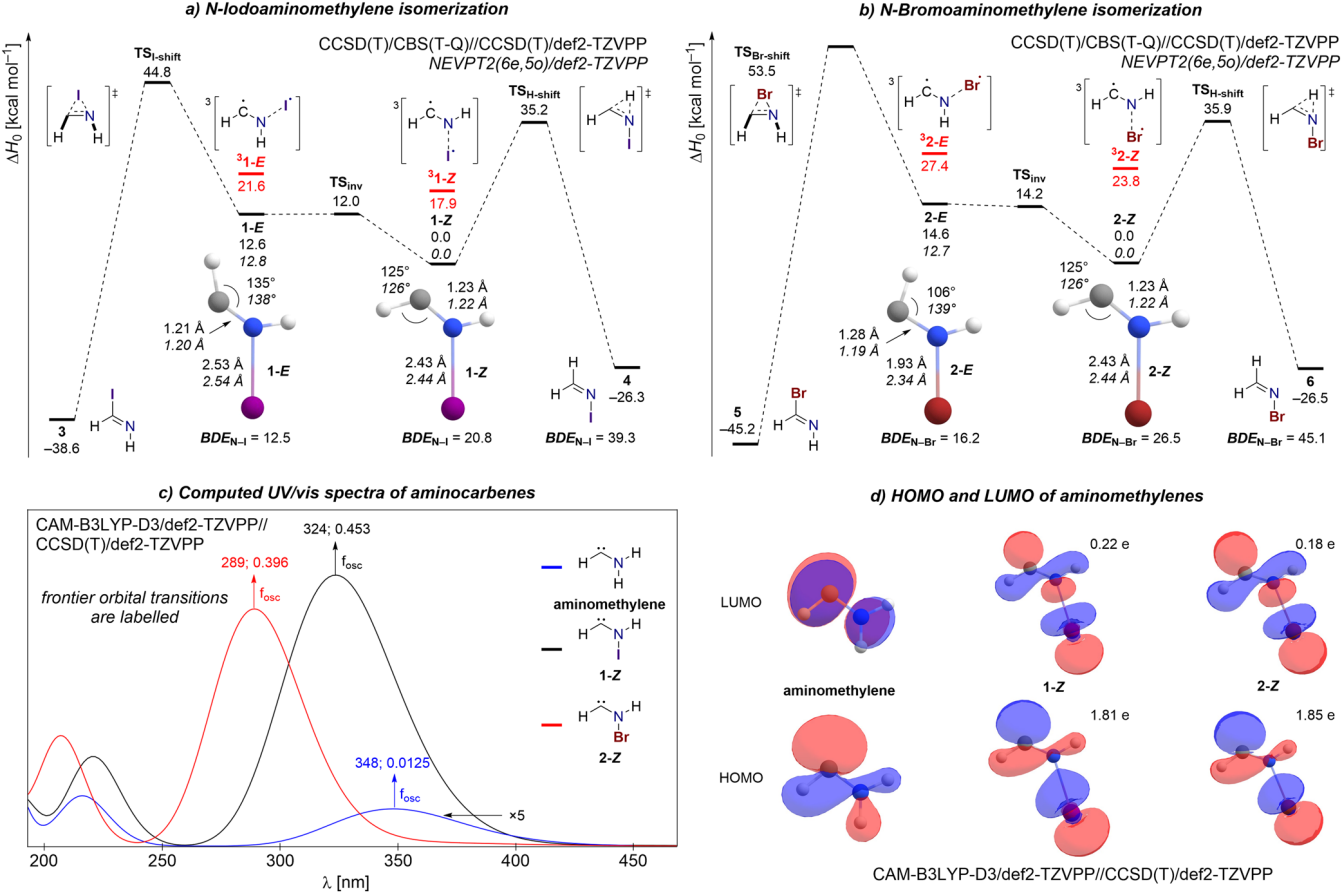
Potential energy surfaces of the isomerization of **1** (a) and **2** (b). Computed TD‐DFT spectra of the aminocarbenes. The intensity of the aminomethylene spectrum is multiplied by a factor of 5 (c). HOMO and LUMO of aminocarbenes, with natural occupation numbers obtained at the CASSCF/def2‐TZVPP level (d).

Since we focused on the monohalogenated aminomethylenes, these carbenes can exist in the form of an *E‐* and a *Z*‐isomer, the former being the only accessible structure in the previously studied cyclic derivatives [14, 15, 18]. We find that the *Z*‐isomers are strongly preferred by 12.6 and 14.6 kcal mol^−1^ in the case of carbene **1** and **2**, respectively (Figure 2). The inversion transition states (TS_inv_) are essentially isoenergetic with the *E*‐carbenes, rendering these less stable isomers unobservable in a prospective matrix isolation experiment. Attempts to localize a distinct rotational transition state consistently converged to the inversion transition state structure, indicating that rotation does not constitute an independent mechanistic pathway.

Notably, we encountered an inconsistency regarding the structure of carbene **2**‐*E*, for which CCSD(T) does not predict the structural hallmarks of the σ‐resonance, namely a flattened H–C–N angle and a shortened C–N bond. Since these carbenes inherently possess multireference character, single‐reference coupled cluster theory potentially faces limitations in the description of their electronic structure, although the *T*_1_ diagnostic [26] values only slightly exceed the threshold of 0.02. Therefore, we additionally cross‐checked the results utilizing full NEVPT2(6e,5o)/def2‐TZVPP optimizations. While the geometric structures and relative energies largely agree in the case of iodinated aminomethylene **1**, the *E*‐isomer of **2** exhibits a larger carbene angle (139°), a shorter C–N bond (1.19 Å), and an increased N–Br distance (2.34 Å) in good agreement with the expectations for this new class of carbenes.

**SCHEME 1:**
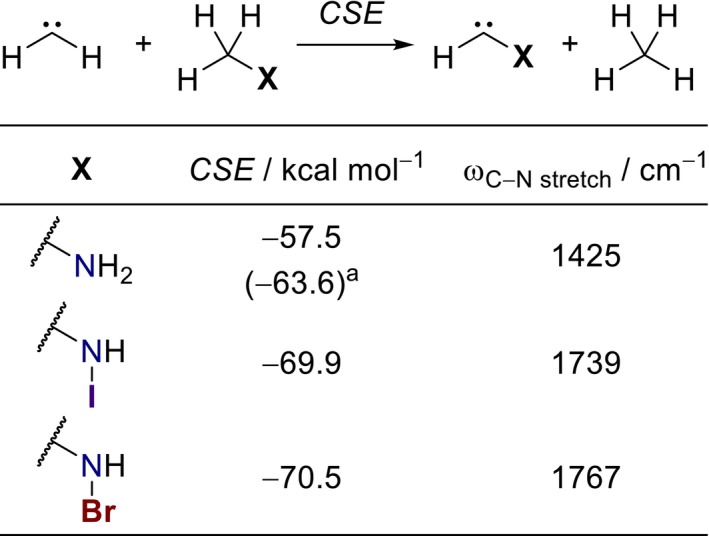
Carbene stabilization energies (CSEs) at the CCSD(T)/CBS(T‐Q)//CCSD(T)/def2‐TZVPP level of theory and harmonic vibrational energies of the C–N stretching modes at CCSD(T)/def2‐TZVPP. ^a^CSE value at the B3LYP/6‐311+G(d,p) level taken from [25].

The optimized *Z*‐isomer structures of carbenes **1** and **2** generally support the notion of strongly stabilizing π‐ and σ‐resonance interactions. In agreement with the expected C≡N triple‐bond resonance contributor (Figure 1b), we find wide H–C–N carbene angles of 125°, significantly different from the analogous angle of 106° in aminomethylene, where no σ‐resonance is available. Additional insight is obtained from comparison of the computed C–N bond lengths with standard values for single, double, and triple bonds [27]. The reported C–N distance in aminomethylene (1.31 Å) falls between those expected for C–N single (1.56 Å) and C═N double bonds (1.27 Å) [25]. In *N*‐halogenated aminomethylenes **1** and **2**, the corresponding C–N bond length of 1.23 Å is shorter than expected for a C═N double bond and approaches the limiting C≡N triple‐bond length (1.14 Å). The concomitant strengthening of the C–N bond is further supported by a blue shift of more than 300 cm^−1^ in the corresponding harmonic stretching vibration compared to the parent aminomethylene (Scheme 1).

To further characterize the σ‐resonance interaction, we performed NBO analyses for both isomers of **1** and **2**. For the *Z* conformers, substantial second‐order perturbation energies were found for donation of the carbene lone pair into the antibonding σ*_N–X_ orbital, amounting to 372 kcal mol^−1^ for X = I and 513 kcal mol^−1^ for X = Br. These large stabilization energies are consistent with a significant population of the σ*_N–X_ orbital and a concomitant weakening of the N–X bond. However, the NBO results for the *E* isomers proved ambiguous and less consistent, especially in the context of a natural resonance theory analysis. This behavior can likely be attributed to the partially multireference character of the electronic structure, which becomes evident in the fractional occupation numbers of the σ and σ*_N–X_ orbitals in carbenes **1** and **2** in a CASSCF theoretical framework (see Figure 2). Because the NBO analysis relies on a single‐reference DFT description, it may provide an incomplete picture for such systems. Consequently, the NBO results should be interpreted qualitatively. Overall, the findings indicate pronounced donation into the σ*_N–X_ orbital, consistent with a significant weakening of the N–X bond. Thus, the BDEs in the carbenes are reduced by roughly 19 kcal mol^−1^ compared to the corresponding imines **4** and **6** (cf. Figure 2), providing clear evidence for a σ‐resonance stabilization.

These results indicate that σ‐resonance stabilization had not yet reached its full potential in the cyclic pyridinylidenes previously isolated in our laboratory [14, 15]. However, the lower‐energy *Z*‐isomers of the halogenated aminomethylenes show a reduced structural manifestation of this stabilizing interaction, as reflected by slightly longer C–N bond lengths compared to the higher‐energy *E*‐isomers (cf. Figure 2). This apparent contradiction is resolved by considering dipole moments as an additional stabilizing factor. The *Z*‐isomers of **1** and **2** exhibit dipole moments of 3.36 and 3.69 D, respectively, which are substantially smaller than those of the corresponding *E*‐isomers (5.29 and 6.02 D), as computed at the NEVPT2 level.

The σ‐resonance interaction evident in the geometries of carbenes **1** and **2** is expected to translate into energetic stabilization. To quantify this effect, we employed the isodesmic equation shown in Scheme 1 to determine the carbene stabilization energy (CSE), corresponding to the difference in hydrogenation energy relative to methylene [28]. Both *N*‐bromo‐ and *N*‐iodo‐substituted aminomethylenes exhibit stabilization of at least 12 kcal mol^−1^ in comparison to aminomethylene, with negligible differences between the halogens. Comparison with a previously reported CSE value obtained at a different level of theory [25] shows some quantitative deviation, but the qualitative trend remains unchanged.

We next assessed the kinetic stability of the proposed carbenes by examining their susceptibility to unimolecular rearrangement; specifically, [1,2]‐hydrogen and halogen shift reactions were considered (Figure 2). Hydrogen migration in **1** and **2** to form *N*‐halogenated imines proceeds with barriers of about 35 kcal mol^−1^, approximately 10 kcal mol^−1^ lower than in the parent aminomethylene but still kinetically prohibitive [25]. Halogen shift reactions are even more strongly hindered, although they lead to the formation of formimidoyl iodide **3** and bromide **5**, respectively, which are thermodynamically more stable than the corresponding H‐shift products.

We next examined the electronic excitation spectra of the halogenated aminomethylenes in comparison to the parent system. The TD‐DFT‐computed electronic transitions of **1**‐*Z* and **2**‐*Z*, shown in Figure 2, differ markedly from those of aminomethylene. In contrast to the parent carbene, both *N*‐halogenated species display a bright and intense band in their computed UV/vis spectra, which can be assigned to a σ–σ* excitation. This observation is consistent with experimental findings for the *N*‐halogenated pyridinylidenes [14, 15].

Similar to *N*‐halogenated Hammick intermediates (X = I, Br) [14, 15], strong σ‐resonance interactions induce a reordering of the frontier molecular orbitals, resulting in LUMOs with pronounced σ*_N–X_ character. Consequently, the LUMO becomes coplanar with the σ‐type lone pair at the carbene center, enabling substantial mixing of orbital character, as illustrated in Figure 2. This strong spatial coincidence and symmetry matching of the frontier orbitals render the σ–σ* excitation particularly allowed, which is reflected in the large oscillator strengths calculated for **1**‐*Z* and **2**‐*Z* (Figure 2). By contrast, in parent aminomethylene, the HOMO and LUMO possess different symmetries, precluding such mixing and leading to markedly weaker electronic transitions (Figure 2).

## 3 Conclusion

Overall, our results confirm that the twofold resonance stabilization previously observed in *N*‐halogenated pyridinylidenes is further enhanced in the open‐chain aminomethylenes. The *N*‐halogenated aminomethylenes are singlet ground‐state carbenes with high barriers to unimolecular isomerization and increased CSEs relative to the parent aminomethylene. Although their experimental realization is highly attractive, it currently remains challenging due to the inherent instability of their potential photochemical precursors (**3** and **5**).

## 4 Methods

The geometry optimizations were done numerically using coupled cluster theory with single, double, and a perturbative treatment of triple excitations [CCSD(T)] [29, 30]. The carbenes were additionally fully optimized with *n*‐electron valence state perturbation theory without the frozen‐core approximation [31] based on a complete active space self‐consistent field (CASSCF) wavefunction [32, 33] with an active space consisting of six electrons in five orbitals. Numerical vibrational frequency computations were used to confirm the nature of the optimized stationary structures. Time‐dependent density functional computations [34, 35, 36, 37, 38, 39, 40] were done using the Coulomb‐attenuating method with the B3LYP functional (CAM‐B3LYP‐D3) [41, 42, 43]. Ahlrichs' def2‐TZVPP basis set [44, 45] was used throughout. Improved electronic energies were obtained by extrapolation to the CBS limit from explicitly computed def2‐TZVPP and def2‐QZVPP single‐point energies [CBS(T‐Q)] utilizing ORCA's implemented extrapolation routine [46, 47]. A natural bond orbital analysis was conducted using the NBO 6 program at the B3LYP‐D3/def2‐TZVPP level of theory [48]. The carbenic resonance structure was explicitly enforced through user‐defined Lewis structure input to override the default NBO Lewis structure assignment. This approach facilitated the examination of the σ‐resonance interaction via a second‐order perturbation theory analysis of the Fock matrix within the NBO basis. The ORCA quantum chemistry package in its 5.0.1 version was used throughout [49], except for TD‐DFT computations, which were executed with Gaussian 16 [50].

## Funding

This work was supported by Boehringer Ingelheim Stiftung.

## Supporting information


**Data S1:** Supporting Information.

## Data Availability

The data that supports the findings of this study are available in the Supporting Information of this article.
