# Supplementary material for: σ‐Resonance Stabilization of Aminomethylene by N‐Halogenation
Source: J Comput Chem. 2026 Mar 29;47(8):e70360. doi: 10.1002/jcc.70360 (PMC13033820; doi:10.1002/jcc.70360)
Supplement: Supplementary file 1 — Data S1: Supporting Information. [file JCC-47-0-s001.pdf]

- Supporting Information -

σ-Resonance Stabilization of Aminomethylene by *N*-Halogenation

Serhii Medvedko[a], Virinder Bhagat[b] and J. Philipp Wagner*[a]

[a]Institut für Organische und Analytische Chemie, Universität Bremen, Leobener Straße 7, 28359 Bremen

[b]Institut für Organische Chemie, Eberhard Karls Universität Tübingen, Auf der Morgenstelle 18, 72076 Tübingen

*Email: jpw@uni-bremen.de

Table of Contents

S1. Optimized Geometries and Energies S2

S2. Selected Time-Dependent DFT Output Sections: S18

S3. Natural Resonance Theory (NRT) Analysis Results. S20

S4. CASSCF Frontier Molecular Orbitals and Occupancies....................S21

S5. Natural Bond Frontier Orbitals and Second-Order Stabilization Energies.........S22

S1. Optimized Geometries and Energies

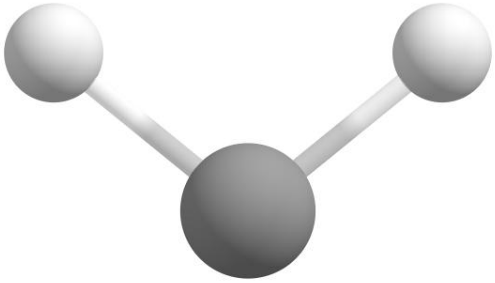

Figure S1. Optimized geometry of singlet **methylene** (in Å) at the CCSD(T)/def2-TZVPP level of theory.

```
1       -0.861093000      0.000000000      0.573395000
6        0.000000000      0.000000000     -0.126698000
1        0.861093000      0.000000000      0.573395000
```

ZPVE (kcal mol^{-1}): 10.44
CCSD(T)/CBS(T-Q) (E_h): –39.078340370700

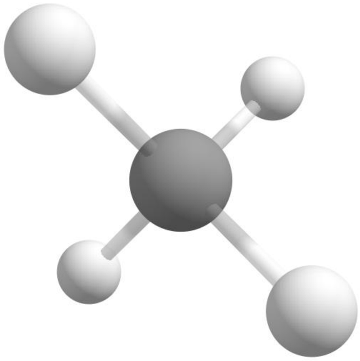

Figure S2. Optimized geometry of singlet **methane** (in Å) at the CCSD(T)/def2-TZVPP level of theory.

```
6        0.000000000      0.000000000      0.000000000
1       -0.628687000      0.628687000      0.628687000
1        0.628687000     -0.628687000      0.628687000
1        0.628687000      0.628687000     -0.628687000
1       -0.628687000     -0.628687000     -0.628687000
```

ZPVE (kcal mol^{-1}): 28.14
CCSD(T)/CBS(T-Q) (E_h): –40.458855516764

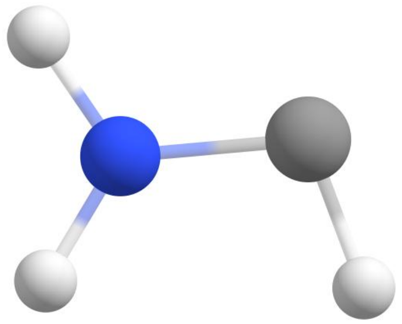

Figure S3. Optimized geometry of singlet **aminomethylene** (in Å) at the CCSD(T)/def2-TZVPP level of theory.

1	0.512701000	2.189927000	0.000000000
6	0.931742000	1.164543000	0.000000000
7	-0.110620000	0.360260000	0.000000000
1	-1.084383000	0.646604000	0.000000000
1	0.035907000	-0.638192000	0.000000000

ZPVE (kcal mol^{-1}): 25.03
CCSD(T)/CBS(T-Q) (E_h): –94.462183841053

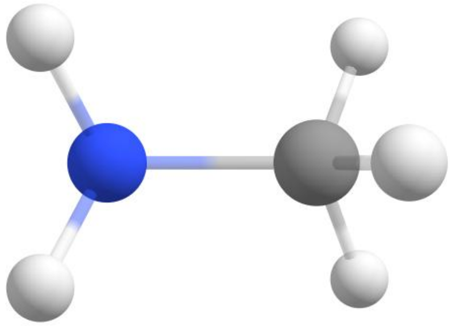

Figure S4. Optimized geometry of singlet **aminomethylene-H$_2$** (in Å) at the CCSD(T)/def2-TZVPP level of theory.

7	-0.124483000	0.351647000	-0.290116000
1	-0.971121000	0.688003000	0.152872000
1	0.083472000	-0.535565000	0.152356000
6	0.970723000	1.295505000	-0.034451000
1	1.184497000	1.479344000	1.025341000
1	1.881025000	0.919947000	-0.503262000
1	0.733386000	2.251463000	-0.502740000

ZPVE (kcal mol^{-1}): 40.37
CCSD(T)/CBS(T-Q) (E_h): –95.747336605628

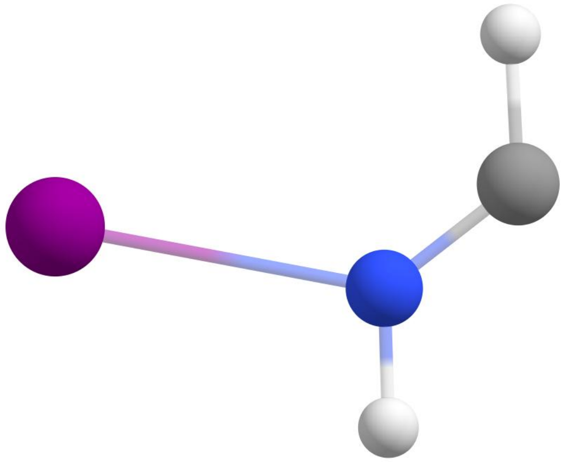

Figure S5. Optimized geometry of singlet **1-*Z*** (in Å) at the CCSD(T)/def2-TZVPP level of theory.

1	1.745134000	1.654135000	0.000000000
6	0.674638000	1.837633000	0.000000000
7	-0.190886000	0.964279000	0.000000000
1	-1.188541000	1.113636000	0.000000000
53	-0.030962000	-1.455693000	0.000000000

ZPVE (kcal mol^{-1}): 18.08
CCSD(T)/CBS(T-Q) (E_h): –391.270086505965

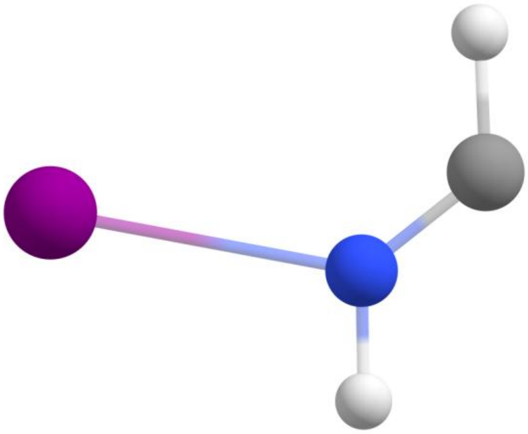

Figure S5A. Optimized geometry of singlet **1-*Z*** (in Å) at the NEVPT2(6e,5o)/def2-TZVPP level of theory.

1	1.743287000	1.659323000	0.000000000
6	0.677690000	1.832884000	0.000000000
7	-0.190265000	0.973730000	0.000000000
1	-1.187561000	1.110165000	0.000000000
53	-0.033768000	-1.462113000	0.000000000

ZPVE (kcal mol^{-1}): 18.23
NEVPT2 (E_h): –391.071924010726

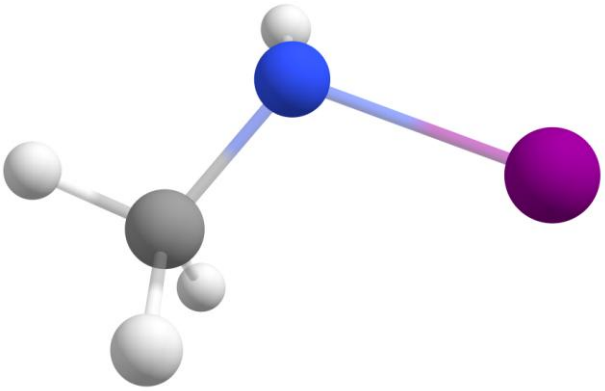

Figure S6. Optimized geometry of singlet **1-*Z*-H₂** (in Å) at the CCSD(T)/def2-TZVPP level of theory.

7	-0.249831000	1.014434000	-0.417582000
1	-1.089640000	1.216248000	0.119426000
53	0.269639000	-0.873741000	0.268288000
6	0.776493000	2.002682000	-0.043896000
1	0.994256000	2.046794000	1.027648000
1	1.697693000	1.773416000	-0.578177000
1	0.417068000	2.979986000	-0.375706000

ZPVE (kcal mol^{-1}): 33.96
CCSD(T)/CBS(T-Q) (E_h): –392.536258053452

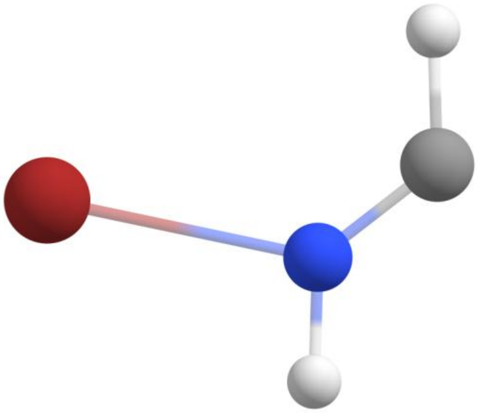

Figure S7. Optimized geometry of singlet **2-*Z*** (in Å) at the CCSD(T)/def2-TZVPP level of theory.

1	1.747783000	1.621479000	0.000000000
6	0.677981000	1.797612000	0.000000000
7	-0.193138000	0.940734000	0.000000000
1	-1.195141000	1.046313000	0.000000000
35	-0.028101000	-1.292148000	0.000000000

ZPVE (kcal mol^{-1}): 18.20
CCSD(T)/CBS(T-Q) (E_h): –2667.134611898566

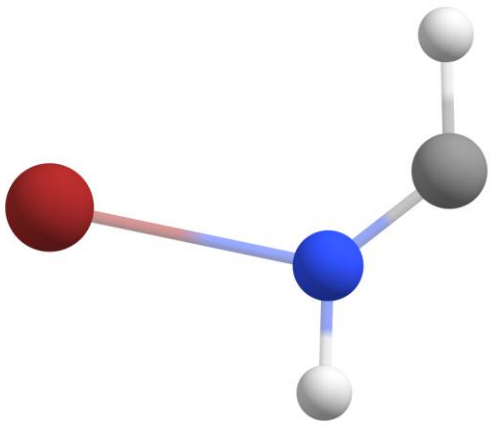

Figure S7A. Optimized geometry of singlet **2-*Z*** (in Å) at the NEVPT2(6e,5o)/def2-TZVPP level of theory.

1	1.743000000	1.623572000	0.000000000
6	0.678399000	1.794759000	0.000000000
7	-0.191754000	0.944470000	0.000000000
1	-1.191969000	1.048581000	0.000000000
35	-0.028293000	-1.297392000	0.000000000

ZPVE (kcal mol^{-1}): 18.77
NEVPT2 (E_h): –2666.582021044681

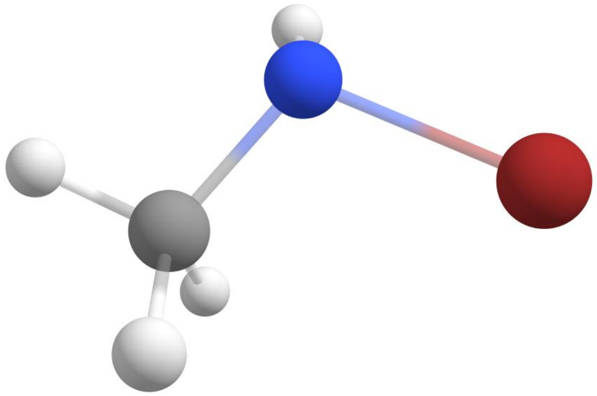

Figure S8. Optimized geometry of singlet **2-*Z*-H$_2$** (in Å) at the CCSD(T)/def2-TZVPP level of theory.

7	-0.277801000	0.997248000	-0.413298000
1	-1.124151000	1.165273000	0.124751000
35	0.291658000	-0.683694000	0.277493000
6	0.704917000	2.028153000	-0.046982000
1	0.911035000	2.086940000	1.025638000
1	1.635555000	1.829689000	-0.576050000
1	0.303245000	2.983907000	-0.391551000

ZPVE (kcal mol^{-1}): 34.26
CCSD(T)/CBS(T-Q) (E_h): –2668.400216812159

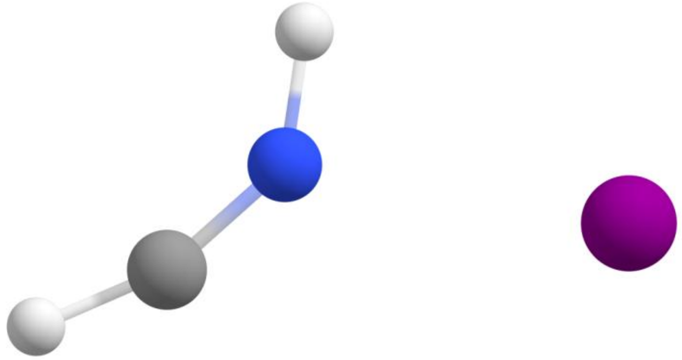

Figure S9. Optimized geometry of singlet **1-TS$_{inv}$** (in Å) at the CCSD(T)/def2-TZVPP level of theory.

1	1.331370000	2.789332000	0.000023000
6	0.778018000	1.873034000	-0.000027000
7	-0.120599000	1.102295000	-0.000006000
1	-1.128729000	1.089650000	0.000000000
53	-0.031047000	-1.515528000	0.000009000

ZPVE (kcal mol^{-1}): 16.31
CCSD(T)/CBS(T-Q) (E_h): –391.248065164286
$\tilde{\nu}_{im}$ (cm^{-1}): –616.27

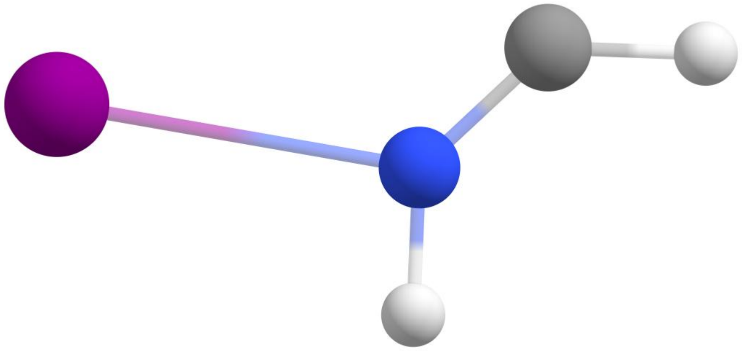

Figure S10. Optimized geometry of singlet **1-*E*** (in Å) at the CCSD(T)/def2-TZVPP level of theory.

1	0.723409000	2.988359000	0.000000000
6	0.689898000	1.905622000	0.000000000
7	-0.189988000	1.081856000	0.000000000
1	-1.198988000	1.108177000	0.000000000
53	0.070248000	-1.430972000	0.000000000

ZPVE (kcal mol^{-1}): 17.26
CCSD(T)/CBS(T-Q) (E_h): –391.248635431180

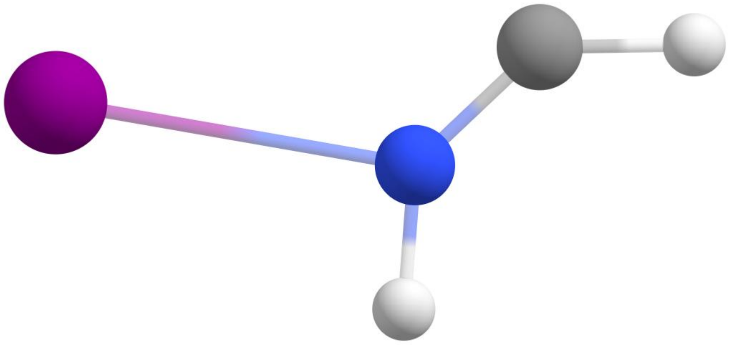

Figure S10A. Optimized geometry of singlet **1-*E*** (in Å) at the NEVPT2(6e,5o)/def2-TZVPP level of theory.

1	0.756110000	2.987430000	0.000000000
6	0.672299000	1.914183000	0.000000000
7	-0.201988000	1.097510000	0.000000000
1	-1.207800000	1.082787000	0.000000000
53	0.075959000	-1.428868000	0.000000000

ZPVE (kcal mol^{-1}): 17.38
NEVPT2(6e,5o) (E_h): –391.050224178434

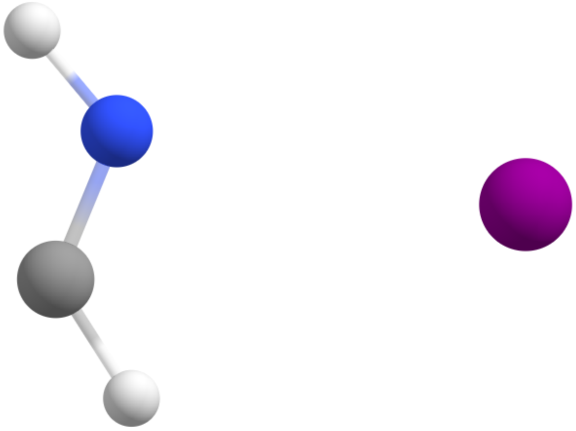

Figure S11. Optimized geometry of triplet **^{3}1-*Z*** (in Å) at the CCSD(T)/def2-TZVPP level of theory.

1	1.639593000	1.261842000	0.000000000
6	0.670814000	1.769631000	0.000000000
7	-0.434108000	1.201409000	0.000000000
1	-1.266689000	1.789194000	0.000000000
53	0.399772000	-1.908086000	0.000000000

ZPVE (kcal mol^{-1}): 16.74
CCSD(T)/CBS(T-Q) (E_h): –391.239400881616

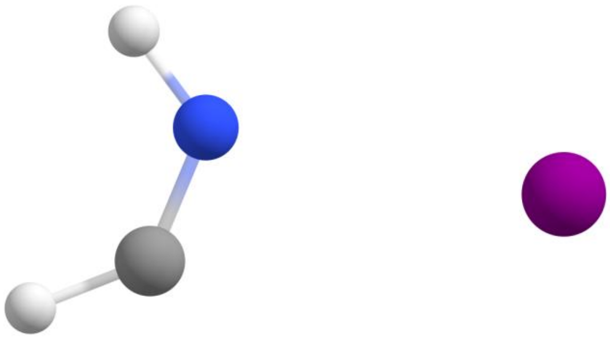

Figure S12. Optimized geometry of triplet **^{3}1-*E*** (in Å) at the CCSD(T)/def2-TZVPP level of theory.

1	0.898797000	2.821172000	0.000000000
6	0.607919000	1.766150000	0.000000000
7	-0.472983000	1.172521000	0.000000000
1	-1.353991000	1.694884000	0.000000000
53	0.414838000	-1.801686000	0.000000000

ZPVE (kcal mol^{-1}): 16.17
CCSD(T)/CBS(T-Q) (E_h): –391.232631345327

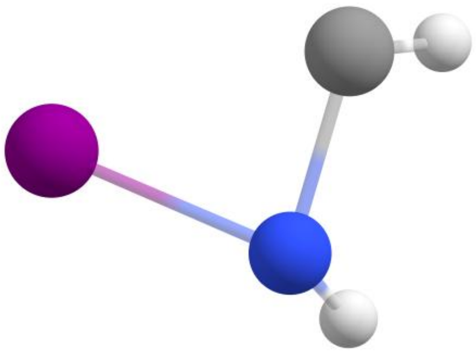

Figure S13. Optimized geometry of singlet **1-TS$_{\text{I-shift}}$** (in Å) at the CCSD(T)/def2-TZVPP level of theory.

1	-2.359485000	-1.100625000	-0.743089000
6	-1.818421000	-0.810069000	0.188336000
7	-1.641148000	0.587854000	0.175105000
1	-2.012243000	1.054660000	-0.664301000
53	0.375654000	0.172453000	-0.176228000

ZPVE (kcal mol^{-1}): 17.08
CCSD(T)/CBS(T-Q) (E_h): –391.197092482253
$\tilde{\nu}_{im}$ (cm^{-1}): –378.24

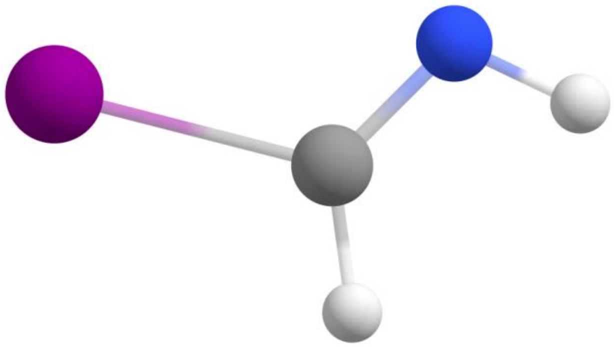

Figure S14. Optimized geometry of singlet **3** (in Å) at the CCSD(T)/def2-TZVPP level of theory.

1	0.477531000	2.945379000	0.000485000
6	0.640221000	1.868986000	-0.001145000
7	-0.240740000	0.963346000	-0.000774000
1	-1.169876000	1.389597000	0.001355000
53	2.682501000	1.377777000	-0.005272000

ZPVE (kcal mol^{-1}): 19.00
CCSD(T)/CBS(T-Q) (E_h): –391.333069905194

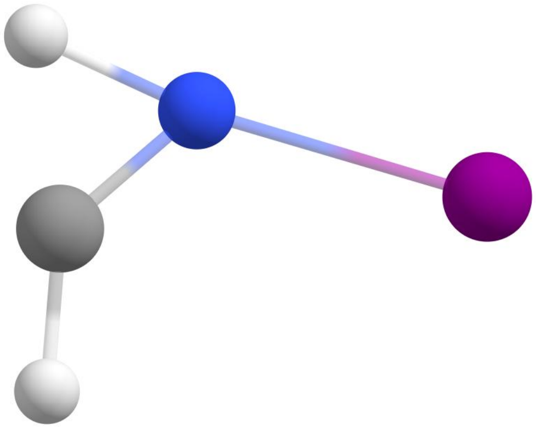

Figure S15. Optimized geometry of singlet **1-TS$_{H\text{-}shift}$** (in Å) at the CCSD(T)/def2-TZVPP level of theory.

1	1.813236000	1.679306000	0.000006000
6	0.724336000	1.778240000	-0.000004000
7	-0.261509000	0.998846000	-0.000024000
1	-0.557080000	2.151514000	0.000015000
53	-0.026983000	-1.377993000	0.000008000

ZPVE (kcal mol^{-1}): 14.12
CCSD(T)/CBS(T-Q) (E_h): –391.207706985361
$\tilde{\nu}_{im}$ (cm^{-1}): –1877.02

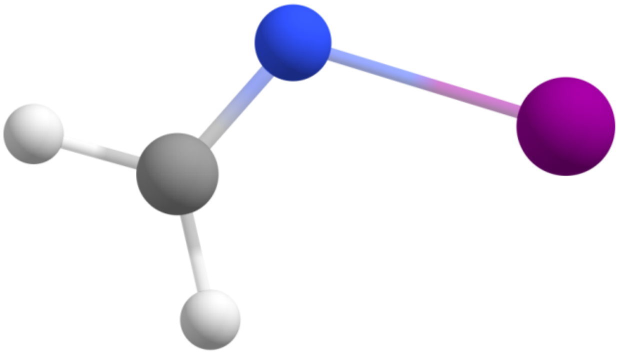

Figure S16. Optimized geometry of singlet **4** (in Å) at the CCSD(T)/def2-TZVPP level of theory.

1	0.683118000	2.645425000	0.000000000
6	-0.400306000	2.533987000	0.000000000
7	-1.039806000	1.428537000	0.000000000
1	-1.012510000	3.434888000	0.000000000
53	0.173949000	-0.263217000	0.000000000

ZPVE (kcal mol^{-1}): 18.61
CCSD(T)/CBS(T-Q) (E_h): –391.312827088891

Energy of doublet **I** (in Å) at the CCSD(T)/CBS(T-Q)// CCSD(T)/def2-TZVPP level of theory.

E_h: –297.380249643336

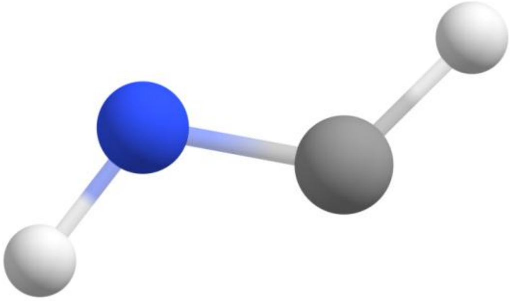

Figure S17. Optimized geometry of doublet **1-*E*-radical** (in Å) at the CCSD(T)/def2-TZVPP level of theory.

1	1.666522000	1.349813000	0.000000000
6	0.673860000	1.808753000	0.000000000
7	-0.396445000	1.180642000	0.000000000
1	-1.257990000	1.725032000	0.000000000

ZPVE (kcal mol^{-1}): 16.26
CCSD(T)/CBS(T-Q) (E_h): –93.853741510904

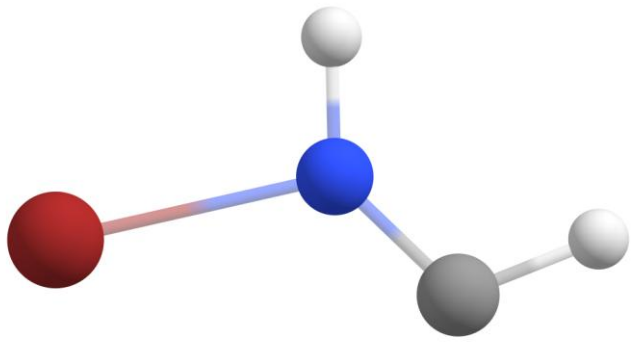

Figure S18. Optimized geometry of singlet **2-TS$_{inv}$** (in Å) at the CCSD(T)/def2-TZVPP level of theory.

1	0.900790000	2.692547000	0.000216000
6	1.035687000	1.606501000	-0.003959000
7	-0.019092000	0.959407000	0.000882000
1	-1.002743000	1.191426000	0.007880000
35	-0.085629000	-1.111098000	-0.005021000

ZPVE (kcal mol^{-1}): 17.35
CCSD(T)/CBS(T-Q) (E_h): –2667.110613675381
$\tilde{\nu}_{im}$ (cm^{-1}): –286.21

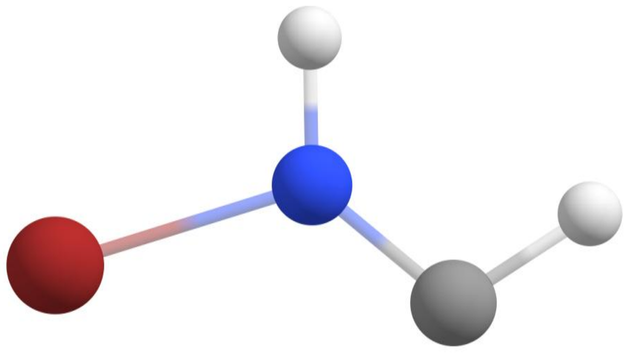

Figure S19. Optimized geometry of singlet **2-*E*** (in Å) at the CCSD(T)/def2-TZVPP level of theory.

1	0.640011000	2.585066000	0.001560000
6	0.980136000	1.534434000	-0.003755000
7	-0.081838000	0.825697000	0.000801000
1	-1.058026000	1.098615000	0.007781000
35	-0.049348000	-1.105898000	-0.005237000

ZPVE (kcal mol^{-1}): 18.07
CCSD(T)/CBS(T-Q) (E_h): –2667.111165765438

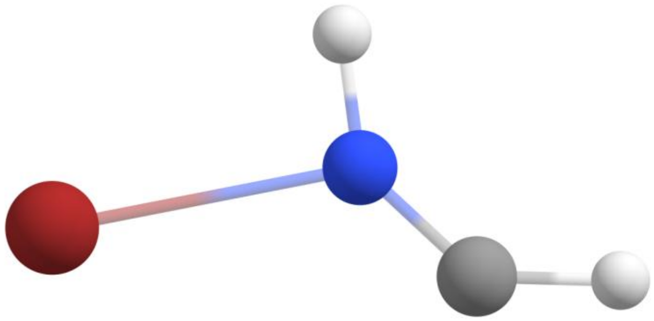

Figure S19A. Optimized geometry of singlet **2-*E*** (in Å) at the NEVPT2(6e,5o)/def2-TZVPP level of theory.

1	0.973563000	2.745037000	0.000316000
6	0.786906000	1.687409000	-0.001824000
7	-0.144275000	0.943494000	0.001454000
1	-1.148522000	0.958281000	0.007566000
35	-0.036736000	-1.396306000	-0.006363000

ZPVE (kcal mol^{-1}): 18.22
NEVPT2 (E_h): –2666.560914506632

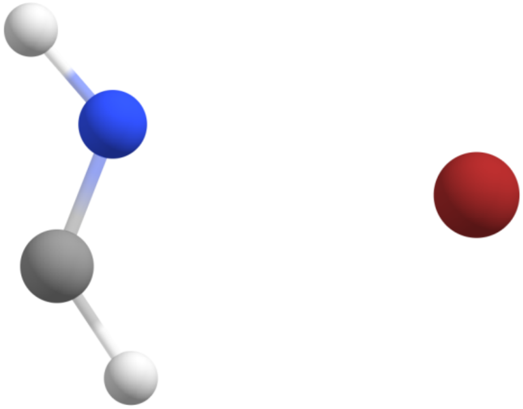

Figure S20. Optimized geometry of triplet **^{3}2-Z** (in Å) at the CCSD(T)/def2-TZVPP level of theory.

1	1.634886000	1.207455000	0.000000000
6	0.670862000	1.723808000	0.000000000
7	-0.438531000	1.164095000	0.000000000
1	-1.267497000	1.756797000	0.000000000
35	0.409665000	-1.738164000	0.000000000

ZPVE (kcal mol^{-1}): 16.75
CCSD(T)/CBS(T-Q) (E_h): –2667.094385442399

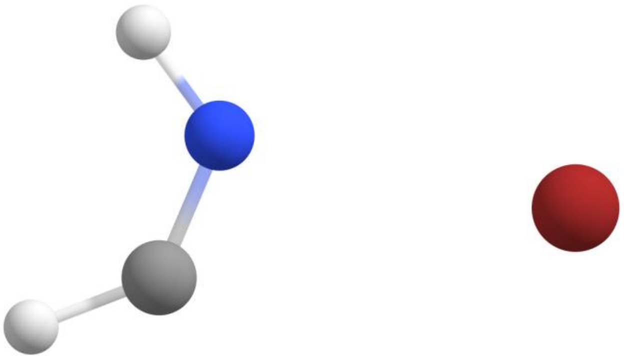

Figure S21. Optimized geometry of triplet **^{3}2-*E*** (in Å) at the CCSD(T)/def2-TZVPP level of theory.

1	0.900249000	2.782359000	0.000000000
6	0.613141000	1.726119000	0.000000000
7	-0.467213000	1.130866000	0.000000000
1	-1.349760000	1.650511000	0.000000000
35	0.398163000	-1.636812000	0.000000000

ZPVE (kcal mol^{-1}): 16.19
CCSD(T)/CBS(T-Q) (E_h): –2667.087704515089

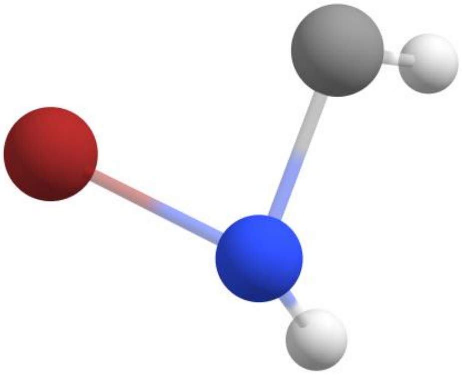

Figure S22. Optimized geometry of singlet **2-TS$_{\mathbf{Br\text{-}shift}}$** (in Å) at the CCSD(T)/def2-TZVPP level of theory.

1	-2.287488000	-1.095347000	-0.755877000
6	-1.813621000	-0.821757000	0.217386000
7	-1.616996000	0.600327000	0.194857000
1	-1.948907000	1.041861000	-0.671471000
35	0.211367000	0.179188000	-0.205073000

ZPVE (kcal mol^{-1}): 17.32
CCSD(T)/CBS(T-Q) (E_h): –2667.048006250106
$\tilde{\nu}_{im}$ (cm^{-1}): –354.51

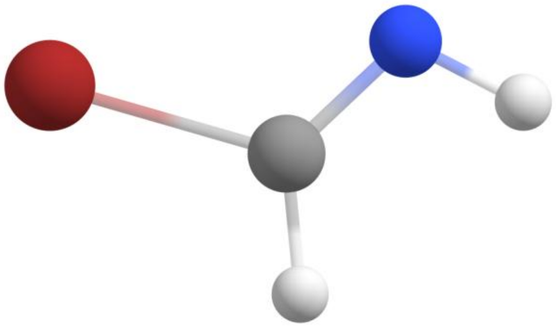

Figure S23. Optimized geometry of singlet **5** (in Å) at the CCSD(T)/def2-TZVPP level of theory.

1	0.526323000	2.935326000	0.000391000
6	0.676459000	1.858449000	-0.001226000
7	-0.203999000	0.955176000	-0.000846000
1	-1.129417000	1.386493000	0.001282000
35	2.520274000	1.409641000	-0.004952000

ZPVE (kcal mol^{-1}): 19.37
CCSD(T)/CBS(T-Q) (E_h): –2667.208557226247

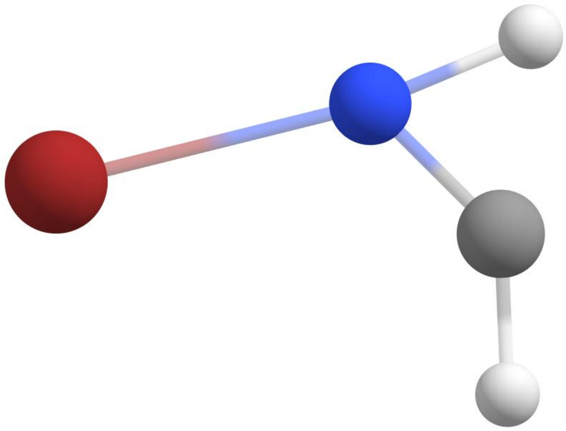

Figure S24. Optimized geometry of singlet **2-TS$_{\text{H-shift}}$** (in Å) at the CCSD(T)/def2-TZVPP level of theory.

1	1.813305000	1.640877000	-0.000005000
6	0.725722000	1.734311000	0.000008000
7	-0.262990000	0.966830000	0.000026000
1	-0.575272000	2.107129000	-0.000022000
35	-0.008765000	-1.219233000	-0.000008000

ZPVE (kcal mol^{-1}): 14.28
CCSD(T)/CBS(T-Q) (E_h): –2667.071134540825
$\tilde{\nu}_{im}$ (cm^{-1}): –1828.41

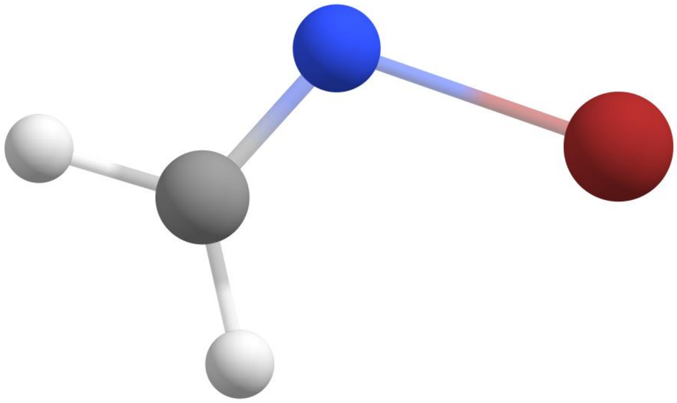

Figure S25. Optimized geometry of singlet **6** (in Å) at the CCSD(T)/def2-TZVPP level of theory.

```
1        0.693300000      2.607740000      0.000000000
6       -0.389145000      2.502159000      0.000000000
7       -1.022480000      1.393092000      0.000000000
1       -1.010396000      3.394279000      0.000000000
35       0.133166000     -0.117650000      0.000000000
```

ZPVE (kcal mol^{-1}): 18.86
CCSD(T)/CBS(T-Q) (E_h): –2667.177821949544

Energy of doublet **Br** (in Å) at the CCSD(T)/CBS(T-Q)// CCSD(T)/def2-TZVPP level of theory.

E_h: –2573.235584268913

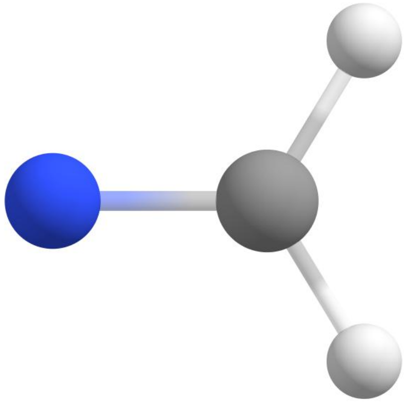

Figure S26. Optimized geometry of doublet methylene amidogen radical (in Å) at the CCSD(T)/def2-TZVPP level of theory.

7	0.000000000	0.000000000	-0.551659000
6	0.000000000	0.000000000	0.700946000
1	0.936869000	0.000000000	1.267776000
1	-0.936869000	0.000000000	1.267776000

ZPVE (kcal mol^{-1}): 15.80
CCSD(T)/CBS(T-Q) (E_h): –93.865541295981

S2. Selected Time-Dependent DFT Output Sections:

S2.1. **Aminomethylene**

```
Excitation energies and oscillator strengths:

 Excited State   1:      Singlet-A"     3.5613 eV  348.14 nm  f=0.0125  <S**2>=0.000
       8 ->  9         0.70753          HOMO -> LUMO
 This state for optimization and/or second-order correction.
 Total Energy, E(TD-HF/TD-DFT) =  -94.4349345650
 Copying the excited state density for this state as the 1-particle RhoCI density.

 Excited State   2:      Singlet-A'     5.7351 eV  216.19 nm  f=0.0169  <S**2>=0.000
       8 -> 10         0.69998

 Excited State   3:      Singlet-A'     6.8656 eV  180.59 nm  f=0.0418  <S**2>=0.000
       8 -> 11         0.69717

 Excited State   4:      Singlet-A'     8.1341 eV  152.42 nm  f=0.1684  <S**2>=0.000
       8 -> 12         0.68834

 Excited State   5:      Singlet-A'     9.0610 eV  136.83 nm  f=0.2415  <S**2>=0.000
       7 ->  9         0.68163

 Excited State   6:      Singlet-A"     9.0721 eV  136.67 nm  f=0.0240  <S**2>=0.000
       7 -> 10         0.70463

 Excited State   7:      Singlet-A"     9.3458 eV  132.66 nm  f=0.0221  <S**2>=0.000
       6 ->  9         0.69842
```

S2.2. **1-*Z***

```
Excitation energies and oscillator strengths:

Excited State   1:      Singlet-A"     2.5852 eV  479.59 nm  f=0.0001  <S**2>=0.000
      19 -> 21         0.70686

 Excited State   2:      Singlet-A'     2.7158 eV  456.53 nm  f=0.0029  <S**2>=0.000
      18 -> 21         0.68870
      20 -> 21        -0.16157

 Excited State   3:      Singlet-A"     3.7499 eV  330.63 nm  f=0.0042  <S**2>=0.000
      20 -> 22         0.70081

 Excited State   4:      Singlet-A'     3.8311 eV  323.63 nm  f=0.4532  <S**2>=0.000
      18 -> 21         0.15790
      19 -> 22        -0.16988
      20 -> 21         0.66560          HOMO -> LUMO
      20 <- 21        -0.13866
 This state for optimization and/or second-order correction.
 Total Energy, E(TD-HF/TD-DFT) =  -391.527595281
 Copying the excited state density for this state as the 1-particle RhoCI density.

 Excited State   5:      Singlet-A"     5.5346 eV  224.02 nm  f=0.0004  <S**2>=0.000
      18 -> 22         0.70499

 Excited State   6:      Singlet-A'     5.6152 eV  220.80 nm  f=0.1520  <S**2>=0.000
      19 -> 22         0.68333
      20 -> 21         0.16405
```

S2.3. **2-*Z***

```
Excitation energies and oscillator strengths:

Excited State   1:      Singlet-A"     3.1716 eV  390.92 nm  f=0.0000  <S**2>=0.000
      24 -> 26         0.70498

 Excited State   2:      Singlet-A'     3.3262 eV  372.75 nm  f=0.0046  <S**2>=0.000
      23 -> 26         0.68702
      25 -> 26        -0.16551

 Excited State   3:      Singlet-A"     3.9692 eV  312.36 nm  f=0.0048  <S**2>=0.000
      25 -> 27         0.69975

 Excited State   4:      Singlet-A'     4.2895 eV  289.04 nm  f=0.3964  <S**2>=0.000
      22 -> 27         0.10165
      23 -> 26         0.16191
      24 -> 27        -0.21452
      25 -> 26         0.64644          HOMO -> LUMO
      25 <- 26        -0.11915
 This state for optimization and/or second-order correction.
 Total Energy, E(TD-HF/TD-DFT) =  -2668.07468009
 Copying the excited state density for this state as the 1-particle RhoCI density.

 Excited State   5:      Singlet-A"     5.8501 eV  211.94 nm  f=0.0003  <S**2>=0.000
      23 -> 27         0.70577

 Excited State   6:      Singlet-A'     5.9845 eV  207.18 nm  f=0.1847  <S**2>=0.000
      24 -> 27         0.66920
      25 -> 26         0.20419
```

S3. Natural Resonance Theory (NRT) Analysis Results.

S3.1. **1-*E***

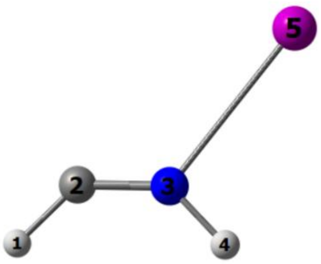

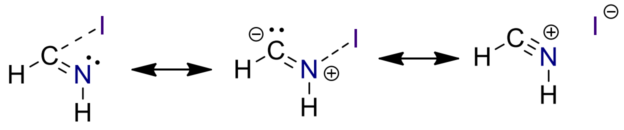

1* (34%) **2*** (30%) **3*** (25%)

S3.2. **1-*Z***

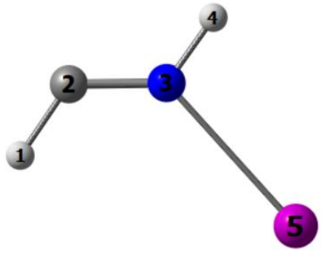

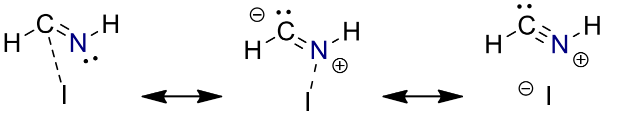

1* (49%) **2*** (26%) **3*** (22%)

S3.3. **2-*E***

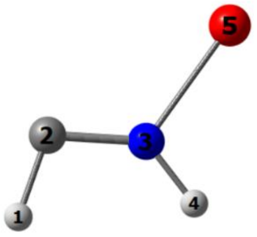

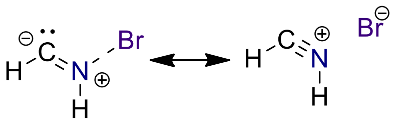

1* (84%) **2** (5%)

S3.4. **2-*Z***

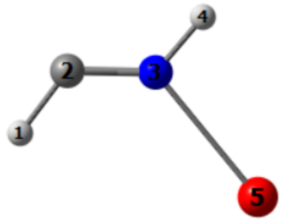

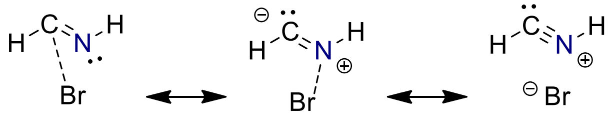

1* (41%) **2*** (32%) **3*** (27%)

S4. CASSCF Frontier Molecular Orbitals and Occupancies

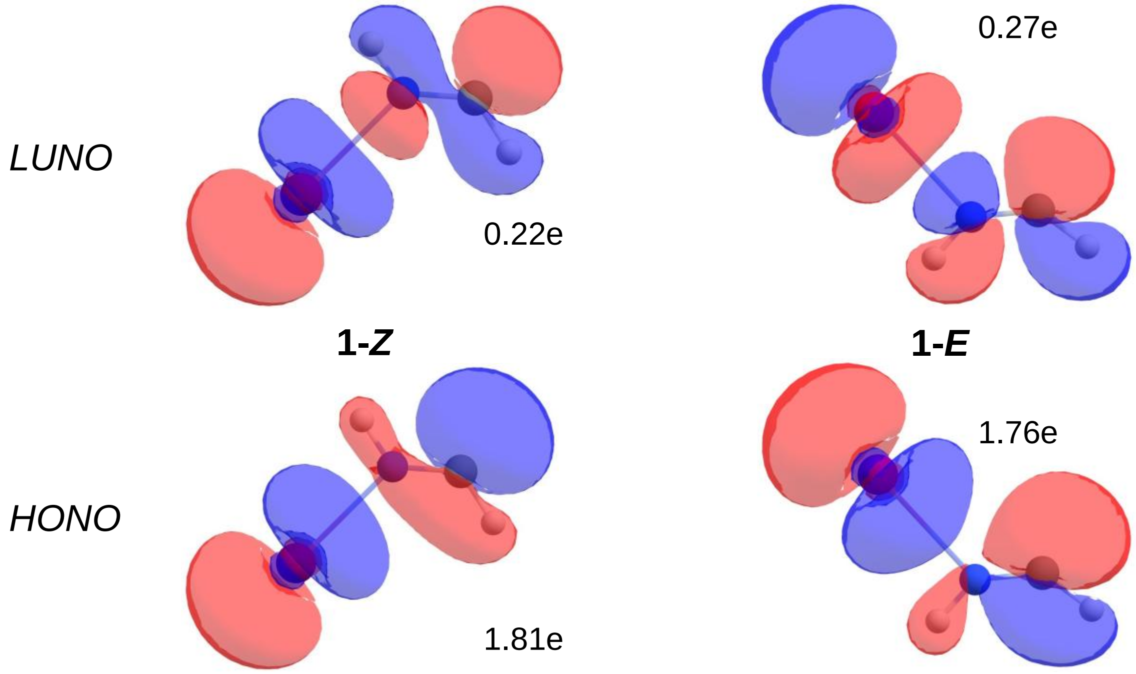

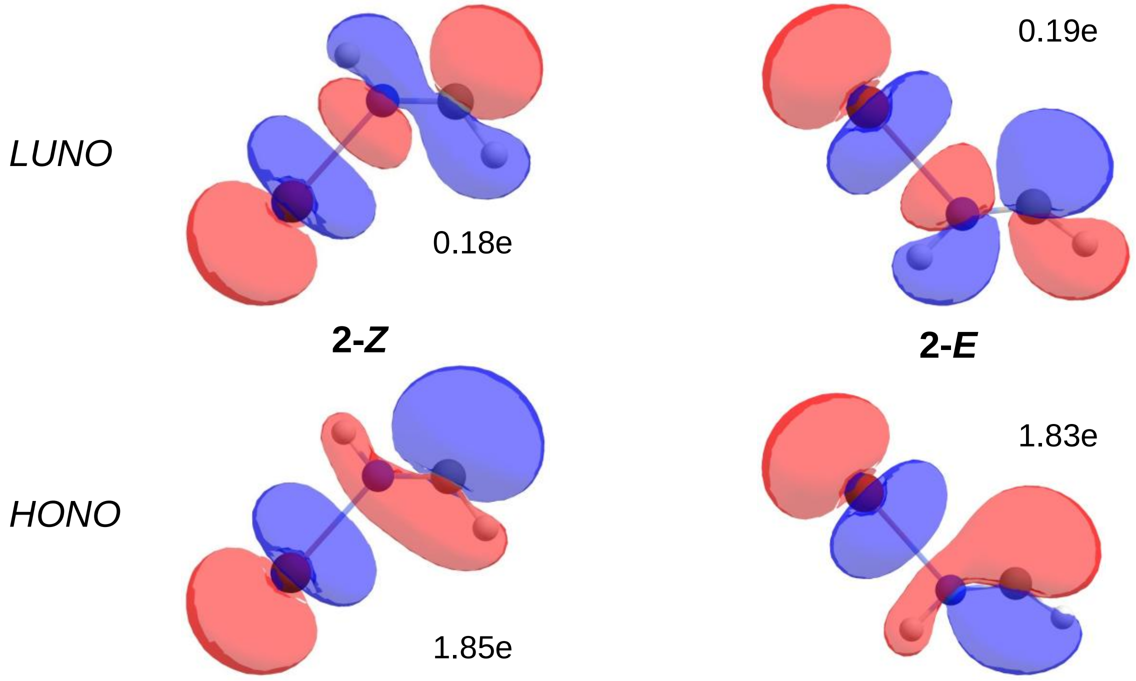

S5. Natural Bond Frontier Orbitals and Second-Order Stabilization Energies

NBO: B3LYP-D3/def2-TZVPP//CCSD(T)/def2-TZVPP

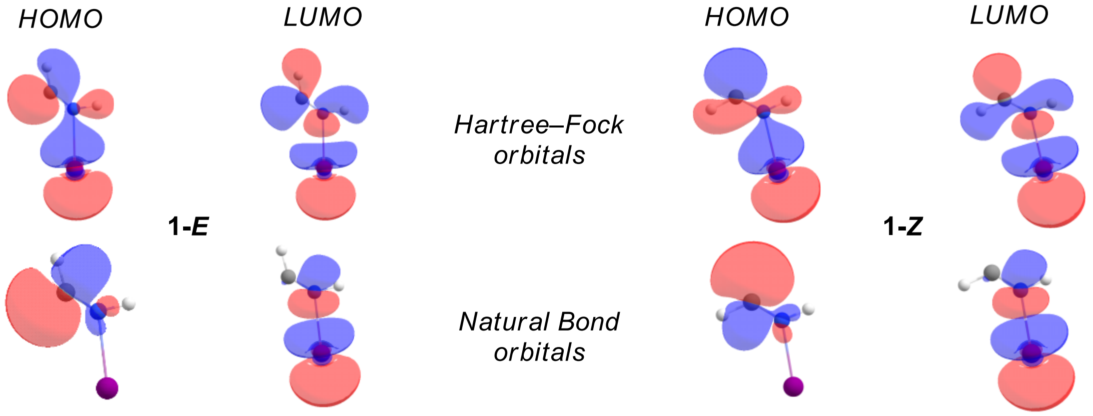

isomer	E(2) kcal/mol [LP_C-$\sigma^*_{N\text{-}I}$]	∠NCH
**1-*E***	not available	135
**1-*Z***	371.63	125

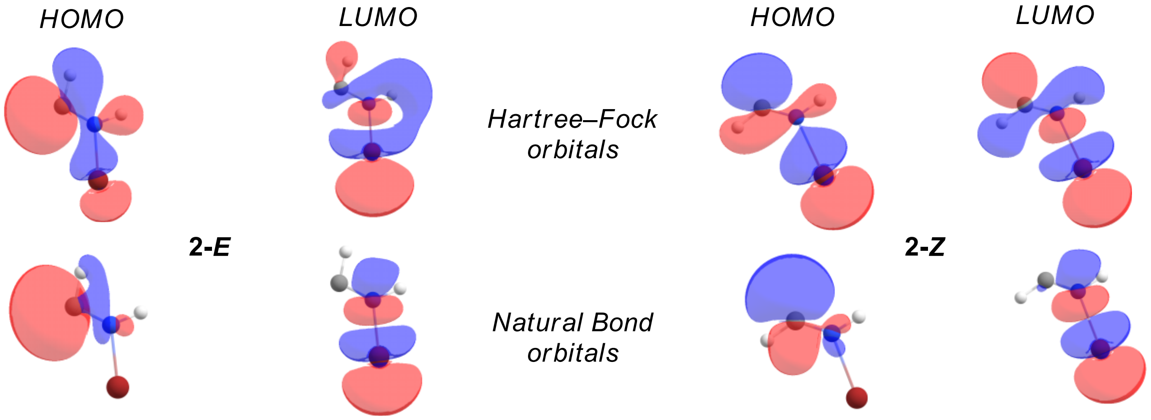

isomer	E(2) kcal/mol [LP_C-$\sigma^*_{N\text{-}Br}$]	∠NCH
**2-*E***	3.23	106
**2-*Z***	513.00	126
